# The experience of mental effort during a continuous performance task: Exploring the influence of task- and person-based factors

**DOI:** 10.1371/journal.pone.0332505

**Published:** 2025-09-26

**Authors:** Danika Wagner, John D. Eastwood

**Affiliations:** Department of Psychology, York University, Toronto, Ontario, Canada; Taipei Veterans General Hospital, TAIWAN

## Abstract

Using mental effort to engage in cognitively demanding tasks is associated with a conscious experience, and this experience serves as a regulatory mechanism. However, important issues remain in our understanding and measurement of the experience of mental effort. For example, essential questions about how person- and task-based factors influence the experience remain unanswered. This study explored how the experience of task-elicited effort and volitionally exerted effort during a continuous performance task (CPT) are associated with person-based (i.e., trait inattention and hyperactivity) and task-based (i.e., manipulations in interstimulus intervals) factors. Participants reported levels of trait inattention and hyperactivity and were randomly assigned to one of three CPT ISI conditions (1000, 3000, or 6000 ms) and provided mid-task ratings of their experience of task-elicited and volitionally exerted mental effort. Both person- and task-based factors were associated with these distinct facets of the experience of mental effort. Several direct relationships exist between trait inattention and hyperactivity, mental effort components, and performance outcomes. However, only one of four moderated mediation models revealed a significant indirect effect: volitionally exerted brain power significantly mediated the relationship between trait inattention and hyperactivity and commission error rates, moderated by task condition with the strongest effect in the 6000 ms ISI. No moderated mediation effects emerged for task-elicited mental effort or latency outcomes. Recognizing that individual differences and task demands result in differing experiences of mental effort which, in turn, predict task performance, is an essential step in tailoring activities and interventions.

## Introduction

The use of mental effort to engage in cognitively demanding tasks is associated with a conscious experience, which regulates the expenditure of cognitive resources [1–3]. However, there is a lack of clarity regarding the conceptualization of mental effort, with theorists emphasizing both task and/or agent aspects (e.g., [4–6]). A recent review underscored the variability in the conceptualizations of mental effort in both theoretical and empirical work and pointed to a distinction between task-elicited (i.e., drawn out by the task) and volitionally exerted (i.e., applied by the agent) mental effort [7]. Moreover, different aspects of the experience of mental effort may vary depending on situational (e.g., [8]) and person-based factors (e.g., [4,9]). Indeed, previous research has shown that participants report different levels of mental effort used for tasks that activate different underlying cognitive processes, such as computational complexity or compensatory control [10,11]. The experience of engaging in mental effort may also differ depending on personality (e.g., [12]) or attentional capabilities (e.g., [13]). Thus, to fully understand how the experience of mental effort regulates the expenditure of cognitive resources, it is critical to measure multiple aspects of the experience of effort and account for how person- and task-based factors alter these experiences. The present study sought to explore how person- and task-based factors predict the experience of two aspects of mental effort (i.e., task-elicited and volitionally-exerted) and how, in turn, these experiences of mental effort predict cognitive performance.

### Conceptualizations of mental effort

Varied conceptualizations of mental effort have been proposed. For example, Kahneman [6] noted that mental effort involves both voluntary (i.e., chosen or applied by the individual) and involuntary (i.e., automatic or engaged by a task) processes. Kahneman [6] emphasized involuntary, task-based factors that influence mental effort while recognizing that the individual also plays a volitional role in the expenditure of cognitive resources. Others define mental effort as an aspect of executive control processes that manage energetic resources [11]. According to Mulder [11], deficient task performance is caused by both person and task-based factors insofar as an individual may be unable to volitionally exert enough effort to maintain optimal arousal when a task is not sufficiently activating. Yet, Mulder’s [11] conceptualization of mental effort focuses primarily on that volitional component. Similarly, Hockey [5] emphasizes a volitional component but conceptualizes mental effort in terms of the interrelation between volitionally exerted and task-elicited components and individual factors such as motivation and fatigue. Finally, Bruya [4] defines mental effort as the subjective experience that arises while engaged in mentally demanding activities that require volitional exertion. Bruya [4] notes activities can be autotelic, for which action and self-awareness merge because the activity provides sufficient stimulus for action, or put differently, the task automatically elicits sufficient activation in the individual [4].

These approaches to understanding and defining mental effort highlight distinct but interrelated components. Mulder’s definition underlines the importance of how the task engages the individual through activation and arousal. This relates to Kahneman’s idea of involuntary mental effort or Bruya’s idea of autotelicity—when a task engages and can activate the individual to meet task demands. However, existing conceptualizations, evident in the work of Hockey [5] and others, also point to a component of effort that is applied voluntarily, such that the focus is on the motivation, actions and goals of the agent. Taken together, existing work highlights the multifaceted nature of mental effort, thus, a complete understanding of mental effort must include both task-elicited and volitionally exerted components.

Given that the conscious experience of engaging in mental effort is thought to play a regulatory role, it is critical to assess this experience directly through self-report. Researchers have assessed the experience of mental effort with self-report scales that are either single-item or multi-faceted. For example, researchers have asked participants to rate their current level of mental effort using single-item scales (e.g., [13–15]). By contrast, multi-faceted scales are designed to assess different aspects of the experience of mental effort. For example, the NASA Task Load Index (NTLX) [16] includes an assessment of ‘frustration,’ and the Dundee State Stress Questionnaire [17] includes an evaluation of ‘stress.’ However, these scales fail to clearly distinguish between volitionally exerted mental effort (i.e., put forth by the individual) and task-elicited mental effort (i.e., elicited from the individual by a task).

Furthermore, self-reports of the experience of mental effort can be assessed either prospectively (e.g., [10]), in the moment (e.g., [13–15]), or retrospectively (e.g., [16–17]). Retrospective measures require participants to reflect on their experience of mental effort after completing a task and are subject to biases like the peak-end effect [18]. The peak-end effect describes a phenomenon in which participants’ retrospective judgments are strongly determined by their ratings of an experience at its peak and the end, and this phenomenon has been observed with mental effort ratings (e.g., [10,13,19,20]). Bambrah et al. [10] found that salient moments of mental effort biased overall retrospective evaluations. Thus, given that the peak-end effect has been observed in the recall of mental effort, it is critical to also examine the experience of mental effort in the moment.

### A new term and an integrative conceptualization of mental effort

Considering the disparate ways ‘mental effort’ has been conceptualized, operationalized, and measured, comparisons across different theorists and studies are fraught with confusion and misunderstanding. Thus, we suggest there is a need for a novel term that is readily understood by research participants and less likely to contain idiosyncratic connotations for participants and researchers. To that end, we propose using the umbrella term *‘brain power’ to refer to the task-elicited and/or volitionally exerted arousal, activation and attention processes required to accomplish mental tasks.* We contend that task-elicited and volitionally exerted processes are consciously perceived and regulate task initiation and persistence.

The State Brain Power Questionnaire (SBPQ) is a new measure of the in-the-moment experience of mental effort designed to be administered mid-task to address existing gaps in the literature [21]. The scale uses the term ‘brain power’ instead of mental effort to avoid potential idiosyncratic understandings of ‘mental effort.’ Unlike existing measurements, the SBPQ captures perceptions of task-elicited and volitionally exerted brain power. Task-elicited brain power is the degree to which the individual perceives that the task activates their mental resources. This captures how stimulating the task feels to the agent, rather than their intentions or choices (e.g., “This task was able to engage my brain power”). Vigilance tasks, for example, do not elicit the levels of activation and arousal within the agent that are needed for successful performance and would be associated with low task-elicited mental effort. High task-elicited brain power ratings reflect the perception that the task strongly activates mental capacity, which could be experienced as facilitative or, if excessive, overwhelming. By contrast, volitionally exerted brain power is the perception of the degree to which an individual must apply their attention and modulate arousal in order to perform a task (e.g., “I had to push myself to use my brain power”). This reflects a self-initiated, agentic process of engaging cognitive capacity, rather than what the task elicits. High volitionally exerted brain power ratings indicate the individual’s perception of deliberate, volitional mental exertion, though this does not necessarily indicate greater efficacy. Indeed, a high level of volitionally exerted effort could reflect the fact that the agent perceives they are not performing well, and thus needs to add ‘something more’ to improve performance. Preliminary research aimed at developing and validating the SBPQ has been conducted. In forthcoming work [21], we have shown that participants can reliably report task-elicited and volitionally exerted brain power while engaged in a task requiring sustained attention and that these components are psychometrically distinct. Using the SBPQ in the present study will allow for the direct assessment of volitionally exerted and task-elicited brain power during a task requiring sustained attention.

### Task-based factors impacting the experience of brain power

The experience of task-elicited and volitionally exerted brain power may systematically differ depending on task-based factors [4,8]. Indeed, previous research has compared individuals’ experience of brain power when completing different types of cognitive tasks [10] or when completing different difficulty levels within the same task [20]. Hsu et al. [20] found that participants reported higher levels of effort, defined as the experience of feeling taxed or burdened, with increasing difficulty levels on a working memory task, while Bambrah et al. [10] found that ratings of in-the-moment volitionally exerted effort were higher when participants completed a task that required compensatory control compared to a task that required computational complexity. These findings highlight that task-based factors may differentially impact the experience of task-elicited and volitionally exerted mental effort.

### Person-based factors impacting the experience of brain power

Previous research has found that the experience of brain power correlates with personality traits, such as conscientiousness. For example, highly conscientious individuals report lower ratings of how hard individuals had to work to accomplish the task (similar to volitionally-exerted effort) and frustration on the NTLX when engaged in cognitively demanding tasks than individuals with low conscientiousness [12]. Additionally, the experience of mental effort and the avoidance of mentally effortful tasks has been identified as an essential area of research within clinical populations [22]. Indeed, mental effort avoidance has been shown to have a transdiagnostic structure associated with traits such as lack of perseverance and impulsiveness [9].

Differences in trait inattention and hyperactivity may be important for understanding how person-based factors predict the experience of brain power. Indeed, a diagnostic criterion for Attention-Deficit/Hyperactivity Disorder (ADHD) includes avoiding or disliking tasks that involve sustained mental effort [23]. In a recent review of the literature exploring the experience of brain power in ADHD, Wagner et al. [7] suggest that individuals diagnosed with ADHD and individuals who self-report ADHD symptoms may not be sufficiently activated by cognitively demanding tasks and, therefore, may need to volitionally exert additional brain power to maintain performance. This finding maps to the theoretical understanding of the mechanism underpinning the behavioural deficits observed in ADHD [24–25]. However, Wagner et al. [7] highlight that existing literature has produced mixed results, mainly stemming from different operationalizations and measurements of the experience of brain power. For instance, Mies et al. [26] measured “effort” with the NTLX, which was defined as the work put forth by the individual, while Hsu et al. [13] measured effort, which was defined as the experience of being taxed or burdened. Moreover, previous studies have typically only assessed the experience of using brain power with retrospective measures [26–27] rather than assessments of the in-the-moment experience of doing a task, which is critical for determining task persistence. Therefore, Wagner et al. [7] recommended measuring the self-reported experience of task-elicited and volitionally exerted brain power while engaged in tasks that involve sustained attention, such as the Continuous Performance Task (CPT) [7,28] (see also [26]).

The CPT has been used to explore differences in performance between individuals diagnosed with ADHD and those who have not been diagnosed with ADHD. More specifically, researchers have manipulated the event rate of stimuli (also called inter-stimulus intervals), which alters the demands on information processing during CPTs (see [25,29] for review). Metin et al. [30] conducted a meta-analysis of studies examining event rate effects using a type of CPT (the Go/No-Go paradigm) in ADHD. They found disproportionate slowing of reaction times (RTs) and higher commission error rates for fast (1250–2300 ms) and slow (4250–8300 ms) event rates in the ADHD sample compared to controls. These findings suggest that both very fast and very slow event rates may create conditions that either overwhelm or fail to sufficiently engage individuals with heightened trait inattention and hyperactivity, resulting in greater performance impairments. Thus, this person-based factor may interact with task-based manipulations to produce performance differences. Building on these findings, the subjective experience of mental effort during CPT performance may also vary depending on the event rate. Specifically, task-elicited effort, which reflects the ability of the task to elicit activation and arousal in the agent, may be highest at faster event rates due to the rapid pace of stimulus presentation. In contrast, as event rates slow and stimuli become less frequent, the task may become less activating and arousing, reducing task-elicited effort. However, slower event rates may require greater volitionally exerted effort in order to maintain sustained attention. Critically, prior research has not examined the in-the-moment subjective experience of mental effort, including both task-elicited and volitionally exerted components. The distinction and measurement of the two components are meaningful; while task-elicited effort reflects the effect of the task, volitionally exerted effort reflects the effect of the agent. These two forms of effort may operate through different mechanisms and may differentially regulate task persistence. Specifically, low task-elicited effort may indicate under-arousal, whereas excessively high task-elicited effort may reflect overstimulation, both of which may reduce persistence and impair performance. Volitionally exerted effort, on the other hand, may capture compensatory mental engagement, but higher levels of volitional effort may signal cognitive strain, which may also reduce persistence and impair performance.

Additionally, despite existing research exploring the interaction between ADHD diagnosis and event rate manipulations, there is a dearth of research exploring how task demands interact with continuous measures of trait inattention and hyperactivity in non-clinical samples and how, in turn, this may predict the experience of brain power. Therefore, measurements of trait inattention and hyperactivity allow for an investigation into how person-based factors interact with event rate manipulations (a task-based factor) to predict the experience of brain power. One previous study measured the in-the-moment experience of mental effort (specifically the experience of being taxed or burdened); however, it did not systematically vary task demands [13]. Yet, previous research has shown that varying task demands may elicit different experiences of brain power (e.g., [27]). By measuring two aspects of the experience of brain power (i.e., task-elicited and volitionally exerted mental effort) and by including measurements of person-based factors and manipulations of situation-based factors, we can build toward a nuanced understanding of the regulatory role of the experience of brain power.

### The present study

In summary, existing literature underscores crucial gaps in our understanding of the experience of brain power. Notably, previous studies exploring the experience of brain power have relied on self-report measures that tend to conflate two conceptually distinct aspects of brain power (i.e., task-elicited and volitionally exerted). Using the SBPQ, which is an in-the-moment, multi-faceted measurement of the experience of brain power, in conjunction with manipulations of event rates and assessments of trait inattention and hyperactivity, will address several critical gaps in the existing literature. The present study explored how the in-the-moment experience of task-elicited and volitionally exerted brain power may vary as a function of trait inattention and hyperactivity and interstimulus interval (i.e., 1000 ms, 3000 ms, and 6000 ms ISIs) during a sustained attention task (i.e., Continuous Performance Task). The present study tested whether person-level differences in trait inattention and hyperactivity predict the experience of in-the-moment mental effort, measured separately as task-elicited and volitionally exerted brain power, and whether this experience, in turn, predicts performance on a sustained attention task. We further examined whether task demands, operationalized as interstimulus interval (ISIs), moderate the relationship between person-level differences in trait inattention and hyperactivity and the experience of mental effort. This approach allows us to explore whether the experience of mental effort functions as a regulatory mechanism that mediates the relationship between attentional traits and performance, and how this dynamic varies across different task contexts. This investigation will allow us to clarify and build upon existing understandings of the experience of mental effort to better understand this critical regulator of cognition.

### Hypotheses

All hypotheses were preregistered on the Open Science Framework (https://osf.io/). To access the preregistration for this study, please visit https://osf.io/zmgny [31]. All hypotheses and results are presented in S1 Table of the Appendix.

### Brain power rating hypotheses

Given that event rate manipulations alter the demands on information processing, we hypothesized that ISI condition would affect the experience of using brain power, such that there would be higher ratings of task-elicited brain power in the 1000 ms than the 3000 and 6000 ms conditions and higher ratings of task-elicited brain power in the 3000 ms than the 6000 ms conditions. Moreover, we hypothesized that there would be higher ratings of volitionally exerted brain power on the 6000 ms condition compared to the 1000 ms and 3000 ms conditions.

Despite some mixed findings in the literature, we hypothesized that trait inattention and hyperactivity would significantly predict ratings of the experience of brain power. Specifically, we hypothesized that higher ratings of trait inattention and hyperactivity would be associated with lower ratings of task-elicited and higher ratings of volitionally exerted brain power, collapsed across ISI conditions.

### Overall moderated mediation model

**Fig 1:**
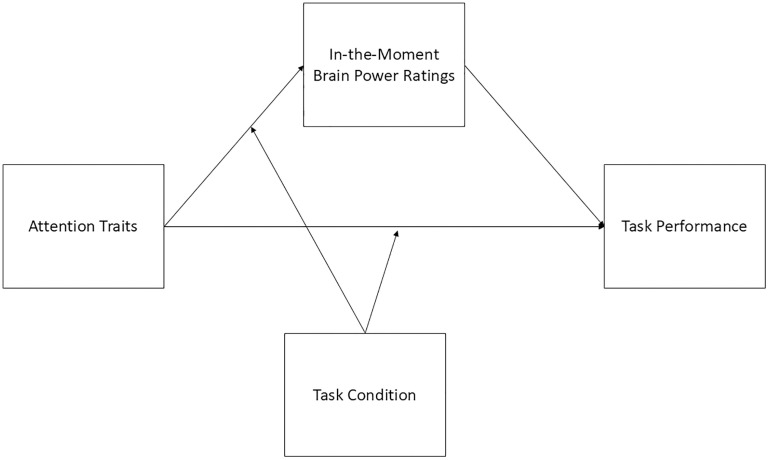
Visual Depiction of the Moderated Mediation Model.

Note. Trait inattention and hyperactivity score = sum of ASRS-5 total and WURS total. In-the-moment brain power ratings = task-elicited or volitionally exerted, run as separate models. Task performance = either commission errors or non-X trial latency, run as separate models. Task condition = 1000, 3000, or 6000 ms ISI.

Building on the hypotheses above, we hypothesized that brain power ratings (i.e., task-elicited, volitionally exerted) will partially mediate the relationship between trait inattention and hyperactivity and CPT performance (commission error rate and non-X trial latency (i.e., non-target trial reaction times)). Specifically, higher attention trait ratings would be associated with lower task-elicited and higher volitionally exerted brain power. We hypothesized that lower task-elicited and higher volitionally-exerted brain power ratings would predict higher commission error rates and slower non-X trial latency. We further hypothesized that task condition (i.e., 1000, 3000, or 6000 ms ISI) would moderate the relationship between trait inattention and hyperactivity and brain power ratings, with larger effects in the 1000 ms and 6000 ms conditions compared to the 3000 ms condition. We also hypothesized a direct path in which higher trait inattention and hyperactivity predict higher commission error rates and slower non-X trial latency, and that this would be moderated by task condition.. See Fig 1 for a depiction of this model.

## Method

### Participants

Participants were students recruited from a metropolitan university in Canada who participated for partial course credit between November 30, 2023 and February 16, 2024. Of the 300 individuals sampled, 79 did not complete the experimental protocol, resulting in a final sample of 221 participants between the ages of 18 and 25 years (*M* = 19.39, *SD* = 1.30). Most participants assigned sex at birth was female (*n* = 183 female, n = 38 male) and identified as women (*n* = 180 women, *n* = 37 men, *n* = 3 prefer not to say). Ninety-one participants had completed high school, 122 had completed some college or university, five had completed a college certificate or university degree, and one had completed a graduate or professional degree. One participant identified as Indigenous Canadian or First Nations, ten identified as Chinese Canadian, 16 as Black Canadian, 22 as English Canadian, 17 as of European descent, 60 as South Asian, eight as West Asian, 48 as “other,” and five preferred not to say. Fourteen participants identified as mixed ethnicity. York University’s Office of Research Ethics approved all study procedures, and informed written consent was obtained from all participants. Data collection and analysis complied with the ethical guidelines of York University and followed all applicable institutional policies and terms of participation.

Table 1 presents mean scores for all 221 participants’ Adult ADHD Self-Report Scale-5 (ASRS-5), Wender Utah Rating Scale (WURS), trait inattention and hyperactivity total score (summed value of the ASRS-5 and WURS), and task performance metrics. To capture a broader and more stable representation of attentional functioning, we combined current (ASRS-5) and retrospective (WURS) measures scores into a single trait inattention and hyperactivity score. This approach provided a more comprehensive estimate of individual differences in attentional traits. There were no significant differences between the three groups in ASRS-5, WURS, or trait inattention and hyperactivity scores. The results pattern showed a ceiling effect for non-X trial accuracy. A summary of the hypotheses and results is in S1 Table in the Appendix. All analyses were conducted using R [32]using the *lavaan* package Version 0.6.18 [33]. A threshold for statistical significance of *α* = 0.05; thus, statistical tests were considered significant at *p* < 0.05.

**Table 1:** Means and Standard Deviations of Background and Outcome Variables.

		Condition	
	1000 ms	3000 ms	6000 ms
Sample Size	68	78	75
ASRS-5 Score	11.15 (4.45)	10.64 (3.83)	11.01 (4.10)
WURS Score	33.91 (21.68)	33.42 (22.50)	32.03 (17.85)
Trait Inattention and Hyperactivity Total Score	45.06 (24.66)	44.06 (24.89)	43.04 (20.57)
CPT Performance			
Non-X Accuracy	0.97 (0.05)	0.96 (0.11)	0.96 (0.09)
Non-X Trial Latency	339.18 (67)	541.00 (331)	830.95 (575)
Commission Error Rate	0.52 (0.25)	0.38 (0.25)	0.39 (0.29)

*Note.* We found a significant difference in commission error rates between the 1000 ms ISI condition and both of the 3000 ms (**p* *= .008) and 6000 ms (*p* = .01) ISI conditions, conditions, *F*(2, 218) = 5.70, *p* = 0.004, differences detected with Tukey’s HSD. No significant difference in commission error rates was found between the 3000 ms and 6000 ms conditions. We also found a significant difference in non-X trial reaction times between conditions, *F*(2, 218) = 28.77, *p* < 0.001. Contrasts between conditions were analyzed using Tukey’s HSD, which revealed faster reaction times in the 1000 ms ISI condition than the 3000 ms (*p* = 0.005) and the 6000 ms condition (*p* < 0.001). A significant difference in reaction times was observed between the 3000 ms and the 6000 ms ISI conditions as well (*p* < 0.001).

### Continuous performance task

The experiment was conducted online using Inquisit 6 Player [34], which presented stimuli on participants’ computers with an average refresh rate of 60 Hz. The present study’s Continuous Performance Task (CPT) version is a Go/No-Go paradigm, also called the non-X CPT. However, the task is manipulated such that participants are randomly assigned to one of three versions of this task. In all versions, participants are presented with a sequence of white sans-serif letters, each for 250 ms, in the centre of a black screen. The difference between the versions of the tasks was the interstimulus intervals (ISI); thus, ISI is a between-subjects manipulation. Three ISIs were used in the present study: 1000 ms, 3000 ms, and 6000 ms. Participants in all task versions were directed to respond quickly and accurately to all letters, except X, by pressing the spacebar. Participants were asked to withhold responding if they saw an X. In all versions of the task, non-X trials occurred 90% of the time and X trials appeared 10% of the time. The frequencies of the two trial types replicate those used in previous studies using the Conners’ CPT [35].

The task began with two practice blocks, each comprising 30 trials matching the 90:10 proportion of the experimental blocks. No feedback was provided after the practice blocks. The practice blocks were followed by two experimental blocks. The first experimental block comprised 100 trials, of which 90 were non-X and 10 were X trials. Following the first experimental block, participants are asked to complete the State Brain Power Questionnaire, described below. Upon finishing the questionnaire, participants completed the second experimental block of trials. This final experimental block included 50 trials, 45 of which were non-X trials and 10 of which were X trials.

### State brain power questionnaire

After the first 100 trials of the CPT, participants completed the State Brain Power Questionnaire (SBPQ) [21]. The SBPQ is a six-item scale that employs the words “brain power” to help overcome the problem of different understandings of the term “mental effort.” The SBPQ addresses two facets of the experience of brain power: (1) how much brain power participants applied volitionally (i.e., volitionally exerted brain power), and (2) how much the task activated their brain power (i.e., task-elicited brain power). Participants indicated their rating of the volitionally exerted (3 items) and task-elicited (3 items) on an 11-point scale from “Only a little” (0) to “A great deal” (10). The SBPQ is presented in the Appendix.

### Adult ADHD self-report scale-5

The Adult ADHD Self-Report Scale (ASRS) is a self-report measure assessing the presence and severity of symptoms related to hyperactivity, impulsivity and inattention in adults within the past six months [36–37]. This six-item scale presents items on a 5-point Likert-type scale from 0 (never) to 4 (very often), with scores ranging from 0 to 24. The responses on the ASRS-5 were summed to create a score of present self-reported trait inattention and hyperactivity.

### Wender Utah rating scale

The Wender Utah Rating Scale (WURS) is a self-report measure assessing the retrospective presence and severity of childhood ADHD symptoms in adult samples [38]. The WURS consists of 25 items on a 5-point Likert-type scale from 0 (not at all/very slightly) to 4 (very much). Scores can range from 0 to 100. The responses on the WURS were summed to create a score of historical trait inattention and hyperactivity.

### Procedure

This experiment was conducted online. After providing electronic written consent, participants completed a demographics questionnaire, the ASRS-5, and the WURS on Qualtrics [39]. They then watched a three-minute video describing our conceptualization of brain power (https://youtu.be/OiSXFuC2yUY). In this video, participants watch animated people perform various tasks, such as mental math or reading. Importantly, this video distinguishes between task-elicited and volitionally exerted brain power. Following the video, participants were forwarded to Inquisit 6 Player [35], where they were randomly assigned to one of three task conditions (1000 ms, 3000 ms, or 6000 ms interstimulus intervals) and completed the CPT and SBPQ. Since participants were randomly assigned to one of three conditions, task condition is a between-subjects factor. Trait inattention and hyperactivity were assessed within subjects, and all participants completed the SBPQ mid-task (mediator) and CPT performance measures (i.e., commission error rates and non-X trial latency; outcome variable).

## Results

### Brain power ratings

To inform the interpretability of the present study, the SBPQ items were submitted to a confirmatory factor analysis with a robust maximum likelihood estimator. A two-factor model indicated acceptable fit to the data: *χ*^2^ (8, *N* = 221) = 22.78, *p* = .004; RMSEA = 0.091, 90% CI [0.049, 0.137]; CFI = 0.987; TLI = 0.976; SRMR = 0.038, AIC = 5470.81, BIC = 5514.98. Internal consistency was high for both SBPQ subscales (task-elicited: *α* = .90, *ω* = .91; volitionally exerted: *α* = .94, *ω* = .95), supporting the reliability of the CFA-derived structure.

To test our hypothesis that brain power ratings would vary across ISI conditions, we conducted two 1x3 ANOVAs. We expected task-elicited ratings to decrease as ISI increased, and volitionally exerted ratings to be highest in the 6000 ms condition. However, we did not find a significant difference in task-elicited brain power ratings between the ISI conditions, *F*(2,219) = 3.45, *p* = .065 (*M*_*1000*_* *= 16.78, *SD*_*10000*_ = 8.03; *M*_*3000*_ = 16.14, *SD*_*30000*_* *= 7.96; *M*_*6000*_ = 14.33, *SD*_*60000*_ = 8.46). We did find, as predicted, a significant difference in volitionally exerted brain power ratings between conditions, *F*(2,218) = 4.28, *p* = .015. Comparisons were analyzed using Tukey’s Honest Significant Difference test, which revealed higher ratings in the 6000 (*M* = 21.01, *SD* = 7.46) ms condition than the 1000 (*M* = 17.5, *SD* = 8.78) ms condition, **p* *= .02, as well as a higher rating in the 6000 (*M* = 21.0, *SD* = 7.46) ms condition than the 3000 (*M* = 17.87, *SD* = 7.82) ms condition, p = .04.

To test our prediction that trait inattention and hyperactivity scores would relate differently to task-elicited versus volitionally exerted brain power, we conducted two regression analyses. First, as predicted, higher self-reported ratings of trait inattention and hyperactivity were associated with lower ratings of task-elicited brain power, *R*^*2*^ = 0.012, *F*(1, 219) = 3.945, *p* = 0.04, *β* = −.047. Second, as predicted, higher self-reported ratings of trait inattention and hyperactivity were associated with higher ratings of volitionally exerted brain power, *R*^*2*^ = 0.011, *F*(1, 219) = 27.46, *p* < .001, *β* = .12.

### Moderated mediation model results

We hypothesized that brain power ratings (i.e., task-elicited, volitionally exerted) would partially mediate the relationship between trait inattention and hyperactivity and CPT performance and that this mediation would differ (i.e., be moderated) by ISI condition. See Fig 1 for a depiction of the overall model, which included nine parameters and was based on data from 221 observations. The figures for each specific model include all the results of the analyses.

### Task-elicited brain power and commission error rate

**Fig 2:**
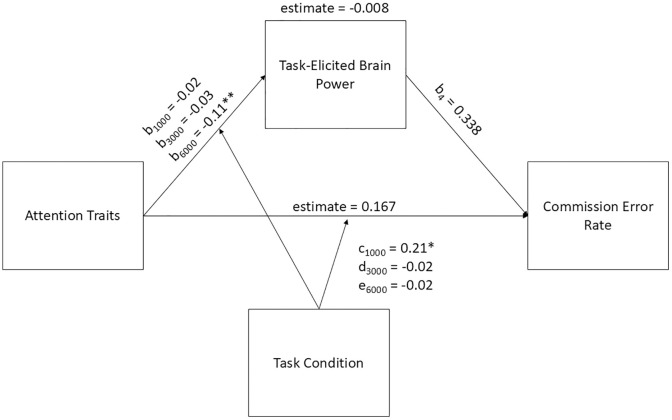
Moderated Mediation Model with Task-Elicited Brain Power as the Mediator between Trait inattention and hyperactivity and Commission Error Rate. Note. * *p* < 0.05, ** indicates p < 0.001. Parameters are as follows: b_1_ and c = 1000 ms ISI condition; b_2_ and d = 3000 ms ISI condition; b_3_ and e = 6000 ms ISI condition.

We hypothesized that higher trait inattention and hyperactivity would predict lower ratings of task-elicited brain power, and, in turn, these lower ratings would predict higher rates of commission errors. We predicted that task condition would moderate the relationship between trait inattention and hyperactivity and task-elicited brain power, with larger effects in the 1000 and 6000 ms condition compared to the 3000 ms condition. Although there were significant paths within the model (see Fig 2), we failed to observe a significant moderated mediation estimate. Thus, the hypothesis for this model was not confirmed.

### Volitionally exerted brain power and commission errors rate

**Fig 3:**
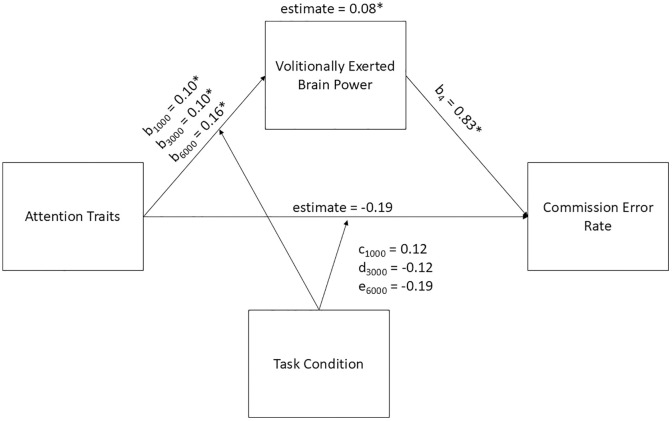
Moderated Mediation Model with Volitionally Exerted Brain Power as the Mediator between Trait inattention and hyperactivity and Commission Error Rates. Note. * indicates *p* < 0.01. Parameters are as follows: b_1_ and c = 1000 ms ISI condition; b_2_ and d = 3000 ms ISI condition; b_3_ and e = 6000 ms ISI condition.

We hypothesized that higher trait inattention and hyperactivity would predict higher ratings of volitionally exerted brain power, and, in turn, these higher ratings would predict higher rates of commission error. We predicted that task condition would moderate the relationship between trait inattention and hyperactivity and volitionally exerted brain power, with larger effects in the 1000 and 6000 ms conditions than the 3000 ms condition. The indirect effect of trait inattention and hyperactivity on commission error rates through volitionally exerted brain power, moderated by task condition, was significant, *estimate* = 0.08, *SE* = 0.03, *z* = 2.64, *p* = 0.008, indicating mediation. The magnitude of the effect was largest in the 6000 ms condition (*b*_*3*_ = 0.16, *p* < 0.01), while the magnitude of the effects in the 1000 (*b*_*1*_ = 0.10, *p* < 0.01) and 3000 (*b*_*2*_ = 0.10, *p* < 0.01), ms conditions were equal. The direct effect of trait inattention and hyperactivity on commission error rate, independent of volitionally exerted brain power, was not significant, *estimate* = −0.19, *SE* = 0.24, *z* = −0.79, *ns,* when ratings of volitionally exerted brain power were included in the model. Overall, these findings confirm the moderated mediation hypothesis, namely, that volitionally exerted brain power mediates the relationship between trait inattention and hyperactivity and commission error rates on a CPT, and the relationship between trait inattention and hyperactivity and volitionally exerted brain power is moderated by task condition. Please see Fig 3 for a visual depiction of the results.

### Task-elicited brain power and non-X trial latency

**Fig 4:**
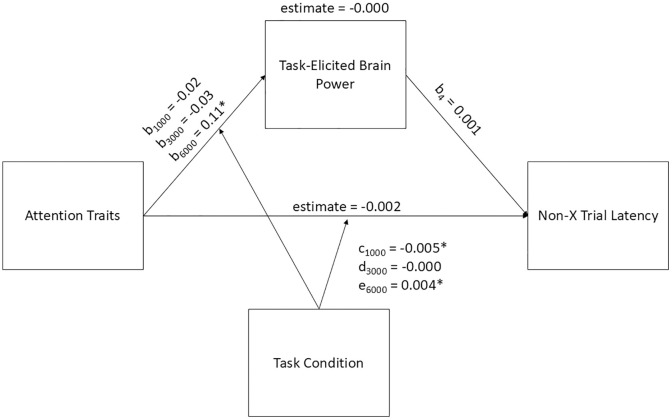
Moderated Mediation Model with Task-Elicited Brain Power as the Mediator between Trait inattention and hyperactivity and Non-X Trial Latency. Note. * indicates *p *< 0.01. Parameters are as follows: b_1_ and c = 1000 ms ISI condition; b_2_ and d = 3000 ms ISI condition; b_3_ and e = 6000 ms ISI condition.

We hypothesized that higher trait inattention and hyperactivity would predict lower ratings of task-elicited brain power, and, in turn, these lower ratings would predict slower reaction times. We predicted that task condition would moderate the relationship between trait inattention and hyperactivity and task-elicited brain power, with larger effects in the 1000 and 6000 ms conditions than the 3000 ms condition. Neither the indirect effect (mediation) of trait inattention and hyperactivity on non-X trial latency through task-elicited brain power, nor the direct effect of trait inattention and hyperactivity on non-X trial latency, independent of task-elicited brain power, was significant. Although there were significant paths within the model (see Fig 4). Thus, the hypothesis for this model was not confirmed.

### Volitionally exerted brain power and non-X trial latency

**Fig 5:**
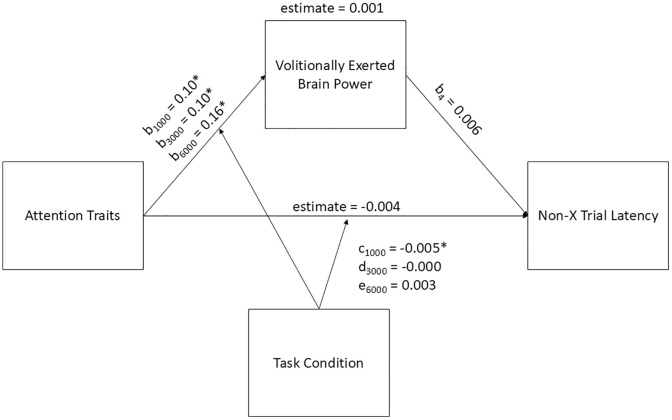
Moderated Mediation Model with Volitionally-Exerted Brain Power as the Mediator between Trait inattention and hyperactivity and Non-X Trial Latency. Note. * indicates *p* < 0.01. Parameters are as follows: b_1_ and c = 1000 ms ISI condition; b_2_ and d = 3000 ms ISI condition; b_3_ and e = 6000 ms ISI condition.

We hypothesized that higher trait inattention and hyperactivity would predict higher ratings of volitionally exerted brain power, and, in turn, these higher ratings would predict slower non-X trial latency. We predicted that task condition would moderate the relationship between trait inattention and hyperactivity and volitionally exerted brain power, with larger effects in the 1000 ms and 6000 ms conditions compared to the 3000 ms condition. The parameter estimates revealed several significant effects within the model, the results of which are presented in Fig 5. Neither the indirect effect (mediation) of trait inattention and hyperactivity on non-X trial latency through volitionally exerted brain power nor the direct effect of trait inattention and hyperactivity on non-X trial latency, independent of volitionally exerted brain power, were significant. Thus, the hypothesis for this model was not confirmed. Please see Fig 5 for a visual depiction of the results.

## Discussion

Although the experience of mental effort is thought to play a critical role in regulating cognition, there is a limited understanding of how person- and task-based factors predict that experience. Moreover, previous research investigating person- and task-based factors has failed to articulate and measure different aspects of the experience of mental effort, which may inform theories of mental effort. The present study sought to explore the experience of task-elicited and volitionally exerted mental effort while accounting for person and task-based factors. More specifically, the present study included scales of trait inattention and hyperactivity as a measure of a person-based factor that is thought to impact the experience of mental effort. Given the scope of our study, several hypotheses were put forward, some of which were confirmed.

First, it was hypothesized that in-the-moment ratings of mental effort would differ depending on task condition and type of brain power measures (i.e., task-elicited or volitionally exerted). We hypothesized that there would be higher ratings of task-elicited mental effort in the 1000 ms condition compared to the 3000 and 6000 ms conditions and higher ratings of task-elicited mental effort in the 3000 ms condition compared to the 6000 ms condition. These hypotheses were not confirmed. Despite preliminary analyses showing that task-elicited mental effort is a psychometrically distinct factor [21], the present study failed to validate our measure of task-elicited mental effort. Although the task-elicited mental effort ratings trended in line with our predictions, manipulations of task factors did not give rise to statistically significant differentiated ratings. It may be the case that the manipulations included in the present study failed to evoke differences in the experience of task-elicited mental effort. Although the present study included a 1000 ms condition, relatively faster than the 3000 and 6000 ISI conditions, event rates on CPTs can be as quick as 500 ms (see [40] for review). Indeed, event rates of less than 1000 ms on a CPT have produced individual differences or symptom-specific performance patterns (e.g., [41]). Therefore, future research should further assess the experience of task-elicited mental effort associated with faster event rates.

We hypothesized and confirmed higher ratings of volitionally exerted effort in the 6000 ms conditions, relative to the 1000 ms and 3000 ms conditions, presumably due to increases in attentional demands at longer ISIs. These findings suggest that the experience of volitionally exerted mental effort is sensitive to task-based factors, particularly when demands are prolonged, as in the 6000 ms condition. Previous research has found that the subjective experience of volitionally exerted mental effort varies depending on task type, with sustained attention tasks experienced as more effortful than working memory tasks [10]. The present study extends previous research by demonstrating that within a sustained attention task, manipulations to temporal structure (i.e., ISI) can meaningfully affect participants’ experience of volitionally-exerted effort.

Second, we hypothesized that self-reported trait inattention and hyperactivity would be associated with differential relationships to task-elicited and volitionally exerted mental effort. We hypothesized and confirmed that higher trait inattention and hyperactivity (i.e., the sum of ASRS and WURS scores) would be associated with lower ratings of task-elicited mental effort and higher ratings of volitionally exerted mental effort when completing a continuous performance task. Previous research exploring the experience of mental effort in ADHD or in association with self-reported symptoms has primarily done so with dichotomous comparisons between an ADHD group and a control group. A recent scoping review found that these studies using dichotomous groups have yielded mixed results in the self-reported experience of mental effort [7]. Only one study has explored the continuous relationship between trait inattention and hyperactivity and the experience of mental effort—Brown et al. [42] found that self-reported trait inattention and hyperactivity accounted for 7% of the variance in self-reported mental effort. However, this study only used a retrospective single-item measure of the experience of mental effort, failing to differentiate between task-elicited and volitionally exerted brain power [42]. The present study provides evidence for a relationship between self-reported trait inattention and hyperactivity and different experiences of mental effort (i.e., task-elicited or volitionally exerted).

Third, we hypothesized that mental effort ratings (i.e., task-elicited and volitionally exerted) would mediate the relationship between trait inattention and hyperactivity and task performance (i.e., commission error rates, non-X trial latency) and that this relationship would be moderated by task condition (i.e., 1000, 3000, 6000 ms). No statistically significant moderated mediation relationships were observed when exploring task-elicited mental effort as the mediator between trait inattention and hyperactivity and either outcome variable (i.e., commission error rates and non-X trial latency). Nor was a statistically significant moderated mediation relationship observed when exploring volitionally exerted mental effort as the mediator between trait inattention and hyperactivity and non-X trial latency. However, volitionally exerted mental effort was found to fully mediate the relationship between trait inattention and hyperactivity and commission error rates, and this relationship was moderated by task condition with larger effects observed in the slowest ISI condition (i.e., 6000 ms ISI).

These results suggest that not all aspects of mental effort or performance outcomes are equally sensitive to trait-level attentional variability or task demands. Volitionally exerted mental effort may better capture compensatory control, particularly evident in individuals with higher attentional difficulties who must actively modulate their cognitive state to sustain task performance. Notably, higher volitionally exerted mental effort was associated with increased commission errors, especially under slower-paced conditions, suggesting that greater volitional investment reflects the subjective difficulty of maintaining engagement. Importantly, the absence of a significant relationship between volitionally exerted effort and non-X trial latency, despite its association with commission errors, suggests that volitionally exerted mental effort may be more strongly linked to the regulation of response inhibition than to general task speed. Whereas non-X trial latency likely reflects sustained attention or general engagement, commission errors require more precise control over impulsive responses. This dissociation underscores that volitionally exerted effort may specifically index compensatory control processes engaged under conditions of cognitive strain, especially in individuals with elevated trait inattention and hyperactivity, rather than uniformly enhancing all aspects of performance. Volitionally exerted effort may serve as an indicator of an individual’s attempts to stabilize task performance through the regulation of arousal and attention processes.

In contrast, task-elicited mental effort did not significantly mediate the relationship between trait inattention and hyperactivity and CPT performance, nor did it exhibit strong variation across task conditions. While task-elicited and volitionally exerted subscales were empirically distinguishable via CFA, this null effect suggests that the automatic, task-driven component of mental effort may not be as impacted by trait attentional characteristics or event rate manipulations as hypothesized. It is possible that task-elicited effort is more stable across individuals or that the current manipulation was insufficient to drive variance in this dimension.

The present findings challenge the common assumption that greater mental effort necessarily translates to better performance. While mental effort is often defined as the investment of cognitive resources and is thus presumed to enhance information processing and task outcomes, our results complicate this notion. Specifically, higher volitionally exerted mental effort was associated with more commission errors, particularly in the slowest-paced condition, suggesting that this form of effort may reflect attempts at compensatory and regulatory processes rather than effective engagement. Individuals with higher attentional difficulties may volitionally invest more mental energy in an attempt to stabilize performance, but this regulation may be insufficient. This aligns with frameworks that distinguish between the amount of effort exerted and its efficacy, and highlights the value of separating subjective, volitionally-exerted mental effort from task-elicited mental effort in understanding the mental effort–performance trade-off [5].

It is commonly recognized that mental effort plays a regulatory role in cognition, the experience of which is strongly associated with negative affect [3,43,44]. Yet, the differentiation between different aspects of the experience of mental effort is often unaccounted for in these understandings. The empirical distinction between task-elicited and volitionally exerted mental effort and the corresponding pattern of results in the present study may allow for new understandings of the experience of mental effort. Indeed, the distinction between task-elicited and volitionally exerted mental effort components has been highlighted previously (e.g., [13,26]) and is borne out empirically. For example, Mulert et al. [45] found that effort required was proportionally linked with objective task difficulty, while volitionally exerted effort (i.e., effort applied) remained stable across difficulty levels. Moreover, Otto et al. [46] found that ratings of volitionally exerted mental effort were correlated with greater activation in the left anterior insular cortex when compared to more task-focused ratings of difficulty. Other researchers have begun to explore similar distinctions between the effort demanded or pulled by the task and the effort volitionally applied and have found that these different aspects of effort are differentially valanced [47]. Thus, the findings from the present study, which found differential effects for task-elicited and volitionally exerted mental effort, provide additional evidence of the multi-dimensional nature of the experience of mental effort, further underscoring the theoretical issues in our understanding of mental effort. Together, these results refine our understanding of how mental effort operates under varying cognitive demands and suggest that volitional and task-elicited components may reflect distinct psychological processes.

No previous research has explored how the in-the-moment experience of mental effort is predicted by person-based (i.e., trait inattention and hyperactivity) and task factors (i.e., event rate manipulations) and how, in turn, this experience is related to cognitive performance. Previous research has found that ADHD-probable individuals differ in their experience of mental effort and perform worse on tasks that require sustained attention compared to controls [13]. Additionally, Hsu et al. [13] found that within the ADHD-probable group, there was a significant negative correlation between in-the-moment ratings of the experience of being taxed or burdened and accuracy. Similarly, Mies et al. [26] explored how adolescents with and without ADHD differ in their retrospective measures of volitionally exerted mental effort across five difficulty levels of a working memory task (n-back). However, they failed to find group differences in mental effort ratings at each task difficulty level [26]. Both Hsu et al. [13] and Mies et al. [26] did not explore if the experience of mental effort mediates the relationship between ADHD group status and task performance. Moreover, Hsu et al. [13] did not measure mental effort using multiple dimensions of the experience. Therefore, the present study builds on the foundational work done by Hsu et al. [13] and Mies et al. [26] by exploring how the experience of mental effort mediates the relationship between a person-based factor (i.e., trait inattention and hyperactivity) and cognitive performance while accounting for how task-based factors (i.e., manipulations in event rate) moderate this relationship.

The experience of mental effort may not have a one-to-one correlation with cognitive processes. Including measures of affective processes, for example, may reveal non-cognitive factors which impact the experience of mental effort. For example, recognizing the role of affective processes has been instrumental in furthering our understanding of pain. According to the Gate Control Theory of pain, affective processes influence the ‘gate,’ or transmission station in the spinal cord, influencing nerve impulse flow to the brain [48]. Thus, the experience of pain is influenced by affective processes based in previous experiences, past conditioning, the meaning attached to pain-producing situations, and catastrophizing [49–50]. Given that mental effort is thought to be aversive, previous experiences, past conditioning, the meaning attached to activities that require brain power, reactivity or catastrophizing, and difficulties in regulating emotions in the face of aversive feelings may be crucial to a full understanding of the experience of mental effort (e.g., [3,43]). Comparable lines of inquiry have been explored in ADHD, which have found that the subjective experience of delay impacts performance over and above explanations based on cognitive processes like inhibition [51]. Moreover, recent meta-analyses have found that effort strongly correlates with negative affect across populations [44]. Thus, affective processes ought to be explored when it comes to understanding the experience of mental effort and the regulation of cognition. By further evaluating additional facets of the experience of mental effort, we can continue to elucidate the experience to build on existing theoretical understandings of it.

When considering how affective and cognitive processes are intertwined and contribute to the experience of mental effort, it is also critical to consider cultural and socio-economic factors that may influence experience. Both socioeconomic background and culture have been found to influence the experience of mental effort. D’Angiulli et al. [52] found that adolescents from a lower socioeconomic context perceived more mental effort in a selective auditory attention task when compared to those from a higher socioeconomic background. Additionally, Widyanti et al. [53] found that culture influenced responses on the NTLX, with Indonesian participants using a narrower range of responses when compared to Dutch participants. More broadly, related traits, such as effortful control (i.e., the ability to regulate impulses and behaviours and motivate action towards a goal) and emotional self-regulation, are impacted by structural factors, such as school and peer environments, family context, socioeconomic context, and ethnic discrimination [54–56]. Therefore, future research should examine structural, cultural, and socio-economic factors that may influence the experience of mental effort and the regulation of cognition.

The findings of this study have significant implications for various settings. In education, it has been established that task-based factors can influence the experience of mental effort. For instance, the difficulty level at the end of a task can predict the recollection of effortful learning [57]. Similarly, positive recollections of learning can be elicited by starting or ending learning with an easy task [19]. Moreover, recent meta-analytic results indicate that higher perceived levels of mental effort is associated with lower confidence in learning, which can have downstream effects on learning outcomes [58]. In the realm of industrial/organizational psychology, studies have shown that employers could reduce employees’ likelihood of resigning by managing the sequence of tasks, such as minimizing the number of hard tasks completed in a row or inter-splicing those hard tasks with easy tasks [59]. The present study’s results demonstrate that individual differences may play a crucial and interacting role with task-based factors. By incorporating assessments of the experience of multiple aspects of mental effort when completing tasks in applied settings and understanding how the experience is shaped by individual differences while considering task factors like sequencing, we can enhance outcomes in diverse domains.

### Limitations

The present study had several limitations. First, previous literature exploring the experience of mental effort has typically included retrospective (e.g., [26]) and/or anticipated (e.g., [10]) measures of the experience. The present study only included in-the-moment measures, which limits the ability to generalize and connect the findings within the larger literature. This choice was deliberate, given the context of the present study. Still, future research should include multiple measurements of the experience of mental effort before, during, and after a task as person and task-based factors may differentially impact them [60]. These factors likely regulate different aspects of behaviour, such as willingness to start a task as opposed to the willingness to persist with an ongoing task.

Another limitation of the present study is the lack of confirmation of the validity of our measure of task-elicited mental effort. This is a crucial aspect, given that the SBPQ was used to explore a moderated mediation between trait inattention and hyperactivity and task performance through the experience of mental effort. Therefore, the failure to confirm one of the basic hypotheses of the present study had implications for the subsequent analyses, namely, the moderated mediation. It is essential to develop a validated measure of task-elicited mental effort and to apply such a measure to a wide range of task types and event rate manipulations.

It is important to note that the present study did not control for time-on-task while including three manipulations in cognitive demands. This is a crucial aspect to consider, as it could influence the observed effects. For instance, all participants completed the same number of trials, but the time spent on the task before rating their experience of brain power varied between conditions. Participants completed their brain power ratings after 2.08, 5.42, and 10.42 minutes for the 1000, 3000, and 6000 ms conditions, respectively. Given that larger effects were observed in the 6000 ms condition, it is possible that what was observed was a time-on-task effect rather than an event rate manipulation effect. Therefore, it is recommended that future research should control time-on-task while manipulating cognitive demands to determine the true nature of the observed effects in the present study.

Finally, our model did not include additional person-based factors beyond trait inattention and hyperactivity, such as conscientiousness—a personality trait known to be inversely related to hyperactivity, impulsivity, and inattentiveness. Although trait inattention and hyperactivity and conscientiousness are conceptually and empirically distinct, they are correlated. Thus, conscientiousness may account for variance in both effort and performance outcomes. Future research would benefit from including conscientiousness and other person-based factors to more fully understand the impact of individual differences in the experience of mental effort and task performance.

## Conclusions

Engaging in challenging tasks that require mental effort is critical for individual and collective flourishing and the experience of mental effort serves to regulate task initiation and persistence. Nevertheless, essential questions about the mechanisms that give rise to the experience of mental effort remain unanswered. The present study sought to answer some of these questions by measuring two aspects of the experience of mental effort and including measures and manipulations of person- and task-based factors, finding that both seem to predict the experience of mental effort. This study reveals that trait inattention and hyperactivity predict the experience of both task-elicited and volitionally exerted mental effort. Moreover, event rate manipulations in the CPT significantly shape the perceptions of volitionally exerted mental effort. The interplay between task- and person-based factors predicts the experience of volitionally exerted mental effort, which, in turn, mediates the relationship between trait inattention and hyperactivity and commission error rates. The present study gives prominence to the idea that the experience of mental effort is multifaceted, and the distinction between different aspects (e.g., task-elicited and volitionally exerted) results in different patterns of results, informing the basis for future theory building.

## Supporting information

S1 Appendix The State Brain Power Questionnaire. (DOCX)

S1 Table Summary table of hypotheses and results. (DOCX)
