# Supplementary material for: The experience of mental effort during a continuous performance task: Exploring the influence of task- and person-based factors
Source: PLoS One. 2025 Sep 26;20(9):e0332505. doi: 10.1371/journal.pone.0332505 (PMC12469259; doi:10.1371/journal.pone.0332505)
Supplement: S1 Appendix — (DOCX) [file pone.0332505.s001.docx]

# **S1 Appendix**

## **State Brain Power Questionnaire**

**Task-elicited questions:**

1. This task was able to engage my brain power:

Only a little A great deal

1. This task was able to energize my brain power:

Only a little A great deal

1. This task stimulated my brain power:
   Only a little A great deal

**Volitional questions:**

1. I had to push myself to use my brain power:

Only a little A great deal

1. I had to force myself to engage my brain power:

Only a little A great deal

1. I needed to push myself to activate my brain power:

Only a little A great deal
