# Supplementary material for: The experience of mental effort during a continuous performance task: Exploring the influence of task- and person-based factors
Source: PLoS One. 2025 Sep 26;20(9):e0332505. doi: 10.1371/journal.pone.0332505 (PMC12469259; doi:10.1371/journal.pone.0332505)
Supplement: S1 Table — (DOCX) [file pone.0332505.s002.docx]

**Table S1**

Hypotheses and Results

| Hypothesis | | | Results |
| --- | --- | --- | --- |
| Brain Power Ratings | | |  |
|  | Task-Elicited Brain Power | |  |
|  |  | Higher ratings of task-elicited brain power in the 1000 ms condition than in the 3000 and 6000 ms conditions. | Not confirmed. |
|  |  | Higher ratings of task-elicited brain power in the 3000 ms condition than in the 6000 ms condition. |  |
|  | Volitionally Exerted Brain Power | |  |
|  |  | Higher ratings of volitionally exerted brain power in the 6000 ms condition than in the 1000 and 3000 ms conditions. | Confirmed. |
|  | Trait Inattention and Hyperactivity and Task-Elicited Brain Power | |  |
|  |  | Higher ratings of trait inattention and hyperactivity will be associated with lower ratings of task-elicited brain power. | Confirmed. |
|  | Trait Inattention and Hyperactivity and Volitionally Exerted Brain Power | |  |
|  |  | Higher ratings of trait inattention and hyperactivity will be associated with higher ratings of volitionally exerted brain power. | Confirmed. |
| Moderated Mediation Models Hypotheses | | |  |
|  | Commission Errors and Task-Elicited Brain Power | |  |
|  |  | Higher trait inattention and hyperactivity predict lower ratings of task-elicited brain power, predicting higher commission errors. Task condition moderates the relationship between trait inattention and hyperactivity and task-elicited brain power, with larger effects in the 1000 and 6000 ms conditions. | Not confirmed. |
|  | Commission Errors and Volitionally Exerted Brain Power | |  |
|  |  | Higher trait inattention and hyperactivity predict higher ratings of volitionally exerted brain power, predicting higher commission errors. Task condition moderates the relationship between attention and volitionally exerted brain power, with larger effects in the 1000 and 6000 ms conditions. | Partially confirmed, larger effect observed in the 6000 ms condition but not the 1000 ms condition. |
|  | Non-X Latency and Task-Elicited Brain Power | |  |
|  |  | Higher trait inattention and hyperactivity predict lower ratings of task-elicited brain power, predicting slower reaction times. Task condition will moderate the relationship between trait inattention and hyperactivity and task-elicited brain power, with larger effects in the 1000 and 6000 ms conditions. . | Not confirmed. |
|  | Non-X Latency and Volitionally Exerted Brain Power | |  |
|  |  | Higher trait inattention and hyperactivity predict higher ratings of volitionally exerted brain power, predicting slower reaction times. Task condition will moderate the relationship between trait inattention and hyperactivity and volitionally exerted brain power, with larger effects in the 1000 and 6000 ms conditions.. | Not confirmed. |
